# The reflective zombie: Problematizing the conceptual framework of reflection in medical education

**DOI:** 10.1007/s40037-018-0479-9

**Published:** 2018-10-23

**Authors:** Anne de la Croix, Mario Veen

**Affiliations:** 1 LEARN! Academy, Vrije Universiteit, Amsterdam, The Netherlands; 2 Research in Education, Amsterdam UMC, VUmc School of Medical Sciences, Amsterdam, The Netherlands; 3 Department of General Practice, Erasmus University Medical Center, Rotterdam, The Netherlands

**Keywords:** Reflection, Methodology, Assessment, Theory

## Abstract

Reflection is an ambiguous and profoundly complex human activity. We celebrate the developments in teaching and researching reflection in education, yet have identified flaws in the way reflection has been operationalized: medical education has translated the age-old concept into a teachable and measureable construct. We fear that in this process of operationalization, the philosophical underpinnings of reflection have been discarded. We illustrate this with a thought experiment about a ‘reflective zombie’: students who have been conditioned to follow prescribed thought steps rather than engaging in truly reflective behaviour. In research and assessment of reflection, measuring tools might be unable to distinguish reflective zombies from students who authentically reflect. We argue that the instrumental approach lies at the root of this problem as it limits the rich concept of reflection and illustrate our point by describing problems related to paradigm (we are looking at reflection in the wrong way), methods (we are using the wrong tools), and epistemics (can we even know what we want to know?). We offer three suggestions for implementing reflection into the curriculum and for research into reflection. First, acknowledge the diversity of reflection and let go of the ‘checklist approach’. Second, embrace the personal nature of reflection by stimulating awareness of one’s personal reflection styles as part of the reflective process. Third, shift the focus of research to the *practice* of reflection. We believe that a strong vision on reflection can lead to a balanced curriculum, setting students up for a lifelong learning as a reflective practitioner.

## Introduction

*Imagine two third year medical students who are identical in almost every way. They wrote identical reflective reports, for which they both received an A−. They both have an IQ of 130, are generally liked by teachers, and their GPA is 3.7. Additionally, they filled in a questionnaire measuring reflective skills, on which they both scored identically. As medical students, they only differ in one aspect: while one of the students has actually reflected, the other just pretended to do so’. How do we know which is which?*

This thought experiment is analogous to Chalmers’ ‘philosophical zombie’, which is an exact physical duplicate of a human being, but without consciousness [1]. The ‘reflective zombie’ is someone who displays all the outer traits of reflection, without having actually reflected. In this thought experiment, the most important clue to distinguish the reflective zombie from the student who has authentically reflected is their score for the written reflective report and the outcome of the questionnaire. However, this does not help us find out who the zombie is. This symbolizes the central problem we address in this paper: an outcome focus, both in measurement and in assessment, has distracted from our understanding of *true *reflection.

The instrumental approach to reflection conditions students to focus on fulfilling the expectations of the assignments, instead of on developing into curious, emotionally intelligent, and critical reflective practitioners. Students know what phrases and expressions to use to emulate reflection [2]. They are good at playing the educational game and at gaming the system to succeed and know, for instance, that ‘they like it if you say you cried’ [3]. Reflective assignments such as portfolios or essays could reward students for doing nothing more than check the boxes of ‘good reflection’, which they can do without *actually* reflecting. This ‘tends toward an individual, non-collaborative, instrumentalist orientation’ [4] and has consequences for the way students navigate their way through medical school. On the other hand, versions of the concept of reflection that do not fit into the prescriptive mould of medical education’s construct of it, are excluded or not seen at all. For example, a student who, after reflecting, concludes that everything is fine just the way it is. This does not fit into the way of thinking in which reflection should always lead to changes in behaviour.

There are many educators who are stimulating students to reflect meaningfully, by holding group conversations [5], or by designing writing tasks that can indeed change ‘the attitudes, values, beliefs, and assumptions of individual participants’ [6]. This is the case for many higher education courses, yet we wish to strengthen these developments by critically elaborating on three flaws in current thinking about reflection in medical education (research) specifically. We join critical calls for a new conceptual approach [2, 7–9], and highlight existing practices that might help us to teach and study reflection in a way that is congruent with its original goals.

## Reflection through the keyhole

Reflection is part of many higher education programs. In this essay, we focus on reflection in medical education. A lot happens in the development of young medical students to medical professionals. They learn to carry great responsibilities for a diverse group of patients, under stress, in multidisciplinary teams, with complex administrative systems. Crucial aspects in this development are the ability to learn from experiences and grow as a professional, and the ability to monitor one’s own thoughts and feelings in relation to the often-challenging circumstances. These abilities are often incorporated into the medical curriculum under the term ‘reflection’ [10].

Most broadly defined, reflection is ‘looking for the meaning of an event, the meaning of a history’ [11]. This can take place in many ways and can involve thought, experiences, emotions, the body, and others. Reflection is generally thought to involve one’s relation to both the inner and outer world. It is about surprise, doubt, and out of the box thinking. There is a fundamental role for emotions—so much so, that the distinction between feeling and thought may be artificial [12]. By introducing the idea of the ‘reflective practitioner’, the field of medical education adopted an age-old concept: Socrates (470–399 BC), Hippocrates (460–370 BC), Aquinas (1225–1274), Hegel (1770–1831), Descartes (1596–1650), and Confucius (551–479 BC) have all eloquently formulated the need for deep thought and reflection.

However, medical education journals give the impression that reflection was invented in 1933, and that all that matters is whether it has three steps or four [13]. Taking its characteristic instrumental approach [14, 15] medical education has translated the complex and rich concept into a construct that is inconsistent with its philosophical underpinnings [8, 9]. Reflection has become an ambiguous notion [16] that can be assessed by checklists, requiring a uniform way of teaching, and guidelines for what counts as ‘proper’ reflection. Medical education research seems preoccupied with three issues: finding a uniform definition or model of reflection, finding a singular way to measure it, and developing a fool proof way of adding reflection to the medical curriculum [10]. As some have noted, this is ironic, given that Dewey conceptualized reflection in education as an antidote to this step-by-step outlining of desired thought processes [16–18].

We fear that certain aspects of teaching and researching reflection in medical education could stimulate zombie-like learning behaviour—which is frustrating for teachers and students alike. The focus on outcomes can lead to ‘poor reflection, lack of engagement from students and low-confidence and apathy of staff’ [19]. We see three key problems with reflection as it is currently approached in medical education (research), to do with paradigms, methods, and epistemics.

## Problems with reflection in medical education

### The paradigm problem

In the middle of the night, a police officer sees a drunken person crawling on the sidewalk under a streetlamp, looking for his lost keys. She offers the drunk man to help him look, and asks where he lost his keys. The man points at a spot 15 yards away. When the officer proposes to look there, the man answers: ‘but there is no light over there’. This famous joke illustrates the ‘streetlight effect’: we tend to look for answers where it is easy for us to see, rather than where they might actually be found [20]. In medical education, knowledge about reflection is sought after in quantitative research, and in terms of measurability. This is not because reflection is necessarily ‘found’ here, but because that is where medical education, with its strong ties to cognitive and behavioural sciences [15], often tends to ‘look for things’. Luckily, qualitative research is slowly more accepted and embraced in medical education.

When the static light beam of medical education shines on reflection, it shows up as a way of thinking that can be broken down into systematically teachable steps. It seems neatly orderable. However, if we turn the beam into a searchlight and shine on actual educational practice, we find that reflection is often messy, unpredictable and intensely personal [5, 21]. But the reality of the classroom—the interactions between teacher and students, and students amongst themselves—is a place where researchers hardly ever look. A different paradigm can open up spaces to look for knowledge about reflection based on how it occurs in practice.

### The methods problem

The translation of reflection into the realm of medical education has brought with it two demands: to measure reflection, and to assess it. These two activities are requirements of medical education, but is reflection itself actually assessable and measurable? There are certainly aspects of reflection that can be counted: number of hours spent on writing an essay, the types of words and sentences used, the extent to which students are rated or rate themselves as empathic (scale 1–5). But ‘not everything that counts can be counted, and not everything that can be counted counts’ [22]. Do the measurable features of reflection tell us anything about its essence?

The sociologist Maslow said: ‘I suppose it is tempting, if the only tool you have is a hammer, to treat everything as if it were a nail’ [23]. We know how to make validated checklists and questionnaires, we know how to draw conclusions from the data we derive from these tools, and we know how to extract publishable information out of these tools. Rich definitions of reflection might not translate to research methods that match its complexity and personal nature. Reflection involves (at least) cognition, emotions, the body, language, consciousness, and experiences. But when we study it, we get out our ‘hammer’ in the shape of psychometrics, and thereby reduce the phenomenon to a series of quantitative measurements. However, it is exactly these measuring tools that eliminate the human nature of reflection, and make it impossible for us to filter out the ‘reflective zombie’. After all, both zombie and authentically reflecting students, would answer ‘yes’ to the question: ‘I take a close look at my own habits of thinking’ from the GRAS reflection questionnaire [24].

### The epistemic problem

At the root of the measurement issue lies an epistemic problem. Reflection is often formulated as a private experience, a ‘silent dialogue between me and myself’ [25]. But if this is true, how can another person judge if I have reflected, and if so, how deeply I reflected? We cannot know another’s innermost thoughts and feelings. For instance, we generally find it unacceptable to tell someone else ‘you are not hungry’ or ‘you don’t have a headache’, because we are unable to access another’s private experience. Yet this is precisely what we ask our educators, and we ask our researchers to score the quality of reflection. This dilemma touches on the basic problem of epistemics: how to access the phenomenon under study. When it comes to ‘territories of knowledge’ [26], or ‘knowables’ [27], one might wonder: who are we to deny that someone has reflected? A student could have the most profound emotional experiences and insights into their professional identity, but might lack the language to express it, or might be so caught up in this process that they do not feel the need to express it.

Access to reflection requires externalization: it is not enough to reflect—one must *demonstrate* reflection. When medical students demonstrate the ‘skill’ of reflection, they must open up private thoughts to observation and assessment. They might prefer to reflect in abstract terms while listening to music, or might reflect by talking about their experiences to friends, yet medical education asks these students to verbalize reflections, which could force students to worry about words, structure, etc. The same issue occurs in research: asking someone to report on their reflection is not a way to measure that original reflection, but is itself an exercise of ‘reflection on reflection’. Externalization changes the very nature of private reflection, as there is now an audience. When we ask students to reflect for their teachers, their reflection will be different from their ‘silent dialogue’. We know students’ learning behaviour is driven by assessment. The reflection will change even more if there are checklists and requirements for ‘good reflection’, possibly resulting in reflective products filled with socially desirable phrases.

Mandatory reflective products can lead to a learning environment in which it is more advantageous for students to demonstrate behaviours that fit the expectations of ‘reflecting’, than it is to invest in an authentic search for meaning. We feel like this view of reflection is needlessly limited and limit*ing* and are worried about a possible zombie apocalypse due to excessive interest in measurement. So, how do we stop it and move forward in a more meaningful way?

## How to prevent zombies?

The rich concept of reflection has been translated into a step-wise process that can easily be assessed and measured within the confines of the medical education paradigm. But this process excludes many other forms of legitimate reflection, and it adds a performative dimension to reflection that makes it impossible to know the difference between authentic reflection and ‘acting reflectively’. This is too bad, because reflection may be the deciding factor if we want students to truly *become* medical professionals rather than merely *acting* professionally [28]. We do not pretend to have all the answers, but are motivated to join the discussion about different ways forward. Existing developments provide exciting and suitable ideas for incorporating reflection in medical education and offer starting points for fostering true reflection in the curriculum. The three most important messages are: accept and embrace diversity in reflection, reflect on reflection, and shift the research focus to the messiness of everyday interactions.

### Embrace diversity, let go of checklists!

If we accept and embrace the diversity of ways in which people reflect, we can move away from a prescriptive focus that defines reflection in advance, and aims to measure or assess. A more informative venture would be to describe all the productive ways in which reflection in medical education can occur. This would also include non-Western practices and philosophies [4]. Since reflection is deeply personal, assessing reflection also requires a personal element. Ironically, current work on reflection aims to exclude subjectivity as much as possible from the assessment process. Laura Shemtob, a medical student from London, eloquently argues the need for a more personalized approach to reflection: ‘Reflective practice has been integrated into my medical education through portfolios, essays, and longitudinal pathways, but my own reflection does not follow any of these templates’ [29].

Rather than pushing every student’s reflection into the same mould, we feel that diversity in reflection needs to be appreciated, and medical school can become the place to talk about personal ways of reflecting. After all, the zombie metaphor we use is not meant as a way of blaming students, but it is a thought experiment to dispute behaviourism. We argue that outer traits which look like reflection do not necessarily point to authentic inner reflection. To think of an assessment system that is more authentic, would require some ‘letting go’ of checklists and uniformity, especially considering the culture of examination in medical education [9]. It requires the recognition that some learning objectives, like reflective ones, might be better left unassessed. Alternatively, we might want to advance the idea of *ipsative assessment* in the area of reflection: assessing the way in which an individual has improved compared to his/her earlier work, or ‘a summative judgement that reflects the learner’s progress rather than outcomes and level of achievement’ [30]. While this is still a form of assessment, it could be used as a way of creating learning conditions that suit individual students’ needs and wishes, and thus creating an encouraging context in which we hope reflection might flourish. We can imagine that students formulate their own learning objectives, based on their preferences on how to reflect. We realize this is an ambitious goal that requires a structural integration of reflection in curricular design and educators who are skilled at coaching students and giving effective feedback.

### Let students reflect on reflection

There is a need to look at reflection differently, and to use different data when studying reflection. Looking for meaning in an event or experience can take on many shapes and forms. We applaud initiatives where students themselves choose a way to share or show reflections, without a predetermined format. We ought to recognize that sharing reflection can be a valuable reflective activity in itself, and not just a means to an end [5, 31]. Luckily, this already happens in many places and could include written reports, audio logs, group discussions, but could also be art, creative writing, meditation, film, poetry, or other ways of expressing oneself [32–35]. These examples show that there is a role for the arts and humanities in medicine [36, 37], though it is not yet widely recognized. This could also prevent reflection becoming overly focusing on the self [8], which can have negative consequences such as rumination, ‘becoming blocked from taking action, loss of spontaneity, pessimism, and falling into a bottomless pit of reflection upon reflection’ [38].

We recommend universities not to stick to one model or protocol, but try different ones and see which are the ones that suit your group and personal teaching style. We do not suggest banning all models of reflection, but to use them pragmatically: ‘All models are wrong, but some are useful’ [39].

Finally, teaching should be focused on creating conditions that foster reflection, rather than trying to teach directly ‘how to reflect’. Strictly speaking, reflection is not something that can be taught, but a human faculty that each of us possesses to some degree, and which can be stimulated. We need to create the best environment for reflection to emerge. According to Driessen [40]: ‘A learning environment and a portfolio that values a reflective dialogue with a trusted person in an open and safe way, is probably the way to go. However, it is a long road for medical education to create such an environment’. We want to motivate students to turn reflection into a lifelong practice, instead of just another assignment they have to complete to get their degree. Therefore, part of the reflection curriculum should be for everyone ‘to work out his or her own system for engaging in reflection’ [41]. Working out which way of reflecting works best for oneself is actually a crucial part of the reflective process. It is necessary if reflection is not to be just one more mandatory exercise, but a gateway to lifelong learning that doctors are able—and motivated—to continue into their professional practice.

### Shift the research focus to the *practice* of reflection

When it comes to future research on reflection, we propose to *de*scribe how reflection actually takes place in practice, rather than *pre*scribe what counts as ‘good reflection’. Studies where students use a personal audio log, for example, can enrich our understanding of workplace learning, simply by describing what students say [42]. As we showed in previous studies, looking at conversational data can show how participants construct their reflection in a group session [5, 21]. We found that groups of doctors in training naturally find their own structure in group reflection sessions, in a way that is not prescribed by their teachers and despite a lack of rules on how to reflect.

We feel there are strong advantages to approaching reflection in medical education as an interactional activity. As mentioned before, formal assessment tools such as checklists cannot establish objectively which of the two students in our thought experiment truly reflected. However, their teachers probably would not need more than ten minutes with either student to get an idea of who truly reflected and who did not. The nature of teachers’ interactions with students, and the role of teachers’ intuition, can unveil more about the students’ reflection than approaches that aim to objectify reflection. What we are trying to say here is that there is a contrast between ‘objectively’ trying to assess reflection and human contact, interaction, and intuition.

You cannot study reflection directly, but you *can* study language and interaction. Whatever feelings and thoughts we share, feature in our conversations as words, gestures, silences and facial expression. Interesting answers can be found in conversations between medical educators and students, between peers, or in study groups. Studies like our previous ones could be used to analyze what reflection is for different people, and data can be used to spark discussions among educators about facilitating reflection and to help teachers create a discursive environment in which reflection can occur. We feel that talk in the educational context can hold interesting answers about the nature of reflection and how students learn from it.

### Reflecting on the reflective zombie

So far, we have focused on the zombie student in our initial thought experiment who did not reflect authentically. But what about the other student, who did reflect and may have experienced benefits and insights from writing the report and filling in the questionnaire? We aimed to suggest ways in which the richness of reflection in medical education can be enhanced—but this does not mean that we should abandon approaches that work, even if they only work for some of the students. For example, there might be some merit in ‘going through the motions’. One of our colleagues stressed this point when talking about becoming a doctor. She did not feel competent and confident when she had just graduated and when she started her practice. She felt like she was faking it. What helped her through were tips and tricks, and some good acting. By doing what she felt was expected of her, she gained insights, and confidence. She learned, she added her own twists, and found her identity as a family doctor—she faked it until she made it. ‘Emotional labour’, the process of displaying emotions that are expected in the workplace, may start out as acting and gradually become authentic [43]. Although ‘deep acting’ is preferred, ‘surface acting’ can play an important role in daily practice. This idea mirrors the thought that one always takes on a certain role in interactions to influence how one is perceived [44]. It bears resemblance to ‘legitimate peripheral participation’, in which newcomers to a specific community of practice behave in a way that is expected in that community, yet they might not have internalized it—it is not yet authentic behaviour [45]. However, it might offer some students a starting point into the journey to having insights about their own behaviour, being open to emotions, and so on. This is also an argument for keeping reflection as a *mandatory *point of the curriculum, despite its drawbacks and potential negative aspects. Making reflection mandatory also communicates to students that reflection is part of their job description, and that it is their individual responsibility to bring this into practice—even if it is impossible for educators to tell them how to do so [46].

## Conclusion

The issues touched upon in this essay are valid for all higher education courses in which reflection has a place, yet focused on medical education. We all want doctors to be critically thinking, emotionally developed and empathic—and above all, reflective. Recent work has shown inspiring and promising approaches to teaching and studying reflection. However, many interpretations of reflection do not do justice to the theoretical underpinnings of reflective thought. As Fendler and colleagues have pointed out [18], if reflection is cast in the same formal terms of the paradigm it is translated into, it incorporates the methodological habits of that paradigm and loses its power to interrupt and question. The tendency to treat reflection as something to measure and to structure contradicts the very nature of reflective thought. The instrumental approach demands that we look for answers where they cannot be found, using tools that do not unlock new insights, making claims about students’ thinking that might not be warranted. A false sense of security may be found in the instrumental approach that leads to extensive checklists, but if this is our sole focus we risk doing the opposite of what reflection is meant to achieve: being genuinely curious and exploring one’s self and one’s experiences, leading to lifelong learning. Reflection is essential to medical education and should be woven into the fabric of the curriculum by acknowledging diversity, fostering a reflective culture, and describing behaviour rather than prescribing.
